# Microfluidic-assisted synthesis and modeling of stimuli-responsive monodispersed chitosan microgels for drug delivery applications

**DOI:** 10.1038/s41598-022-12031-9

**Published:** 2022-05-19

**Authors:** Omid Sartipzadeh, Seyed Morteza Naghib, Fatemeh Haghiralsadat, Farhad Shokati, Mehdi Rahmanian

**Affiliations:** 1 Nanotechnology Department, School of Advanced Technologies, Iran University of Science and Technology (IUST), Tehran, Iran; 2 Medical Nanotechnology and Tissue Engineering Research Center, Yazd Reproductive Sciences Institute, Shahid Sadoughi University of Medical Sciences, Yazd, Iran; 3 Biomaterials and Tissue Engineering Research Group, Department of Interdisciplinary Technologies, Breast Cancer Research Center, Motamed Cancer Institute, ACECR, Tehran, Iran

**Keywords:** Biomedical engineering, Chemical engineering

## Abstract

Droplet microfluidic has been established to synthesize and functionalize micro/nanoparticles for drug delivery and screening, biosensing, cell/tissue engineering, lab-on-a-chip, and organ-on-a-chip have attracted much attention in chemical and biomedical engineering. Chitosan (CS) has been suggested for different biomedical applications due to its unique characteristics, such as antibacterial bioactivities, immune-enhancing influences, and anticancer bioactivities. The simulation results exhibited an alternative for attaining visions in this complex method. In this regard, the role of the flow rate ratio on the CS droplet features, including the generation rate and droplet size, were thoroughly described. Based on the results, an appropriate protocol was advanced for controlling the CS droplet properties for comparing their properties, such as the rate and size of the CS droplets in the microchip. Also, a level set (LS) laminar two-phase flow system was utilized to study the CS droplet-breaking process in the Flow Focused-based microchip. The outcomes demonstrated that different sizes and geometries of CS droplets could be established via varying the several parameters that validated addressing the different challenges for several purposes like drug delivery (the droplets with smaller sizes), tissue engineering, and cell encapsulation (the droplets with larger sizes), lab-on-a-chip, organ-on-a-chip, biosensing and bioimaging (the droplets with different sizes). An experimental study was added to confirm the simulation results. A drug delivery application was established to verify the claim.

## Introduction

Monodisperse micro/nanoparticles with the same morphology and size have attracted much attention in lab-on-a-chip^1,2^, aptasensors^3,4^, biosensors^5–7^, drug delivery^8–10^, tissue engineering^10^, catalysis^11^, and electro/optic devices^12^. Many attempts have been established to generate uniform micro/nano-particles with on-demand and required morphologies, shapes, and sizes by conventional approaches, including dispersion polymerization^13^, emulsion polymerization^14^, precipitation polymerization^15^, layer-by-layer assemblies^16^, and shirasu porous glass (SPG) membrane emulsification^17^. However, the typical emulsion droplet approaches are irrepressible. The generated droplets (particularly the non-spherical) are divergent in shape and size^18^. Due to the interfacial tensions between the two phases, the conventional techniques to produce droplets shrink into spheres, making it hard to provide suitable shaped micro/nanoparticles (with high quality)^9,19^. Furthermore, typical techniques are costly, inflexible, time-consuming, and complex. Thus, superior approaches are required to generate monodisperse micro/nano-particles with on-demand morphology, shape, and size.

Microfluidics and nanofluidics are the sciences and technologies of the systems with integrated channels in micro-scaled and nano-scaled sizes (10 to 100 µm and nm), in which minimal amounts of fluids (generally 1 L to 10^−9^ L) may flow in desired fabrication manipulated and controlled systematically^19^. Droplet microfluidics has attracted much attention in material fabrication and biomedical devices^20^. Droplet microfluidics, including passive hydrodynamic pressure^21^ and active external actuation^22^ techniques, are promising for generating monodisperse micro/nanoparticles. The critical difference is the external forces required for the active method, including thermal, acoustic, pneumatic, magnetic, and electrical. At the same time, microchannel terraces, step emulsification, T-junction, flow-focusing and co-flow are the primary generators for the passive approach^23^. Droplets can be precisely monitored and controlled by the micro/nanomaterial fabrication processes better than typical bulk methods^24^. Droplets generation is tailored by regulating the fluid flow rates and channel geometry^25^. In microfluidic channels, droplets are produced for developing scalable and reproducible micro/nano-particles with fitted and tunable morphologies, shapes, and sizes, which are difficult to reach by conventional bulk approaches^19^.

Polysaccharide micro/nanostructures have increased much attention in various medical and biological applications because of their excellent bioactivity, biodegradability, and cytocompatibility/biocompatibility^26^. CS, a natural polycationic polysaccharide, and its derivatives obtained by deacetylation of chitin, have been extensively applied to drug delivery, tissue engineering, biosensors, biochips, lab-on-a-chip, and cell immobilization and encapsulation^8^. The synthesis approaches of CS micro/nanoparticles with tailored and controllable morphologies and structures have attracted considerable attention in various applications^19^. CS microparticles can be synthesized by different methods, including electrostatic ion gelation^27^, template coating^28^, spray-drying^29^, and complex coacervation methods^30^. However, CS microparticles synthesized by these conventional techniques permanently have apparent drawbacks, including low mechanical characteristics, irrepressible shell thickness, and weak monodispersity^31^.

Moreover, these synthesis approaches occasionally require tedious operating processes that are complicated and arduous. The remaining organic solvent in the synthesis procedure usually restricts their uses in biomedical applications^32^. Due to the precise manipulation of the micro-scaled fluid, microfluidic techniques can flexibly synthesize numerous emulsion droplets with tenable morphologies and structures^33^. Multiple emulsions in microfluidics can synthesize CS microparticles with uniform size and proper monodispersity^34^. Nevertheless, the manipulation and regulations of the microstructure of multiple emulsions need complicated design and fabrication of a fitted microfluidic device, which restricts the scale-up production of microparticles. As a result, more effective and versatile procedures and techniques for synthesizing CS microparticles with tunable particles and structures remain a challenge.

We investigated the microfluidic droplet generation rates and dimensions relating to impressible parameters such as flow velocity and concentration. The COMSOL Multiphysics® 5.4 simulator software has been applied to evaluate these effective parameters. Employing the results of the available investigation, we were able to develop practical microfluidic chips in a customized manner that produced CS-oil-CS double emulsions. Furthermore, we expanded a computational fluid dynamics (CFD)-based model to discern the configuration and features of droplets created in a flow-focusing (FF) microchannel utilizing the Newtonian system. The computer simulation of the CFD model gave a substitute approach to achieve a better understanding of this complicated progression. Subsequently, implementing the program illustrates the importance of two immiscible fluids' physical properties and velocity upon droplets' formation, diameter, and generation rate.

## Attitude

**Figure 1:**
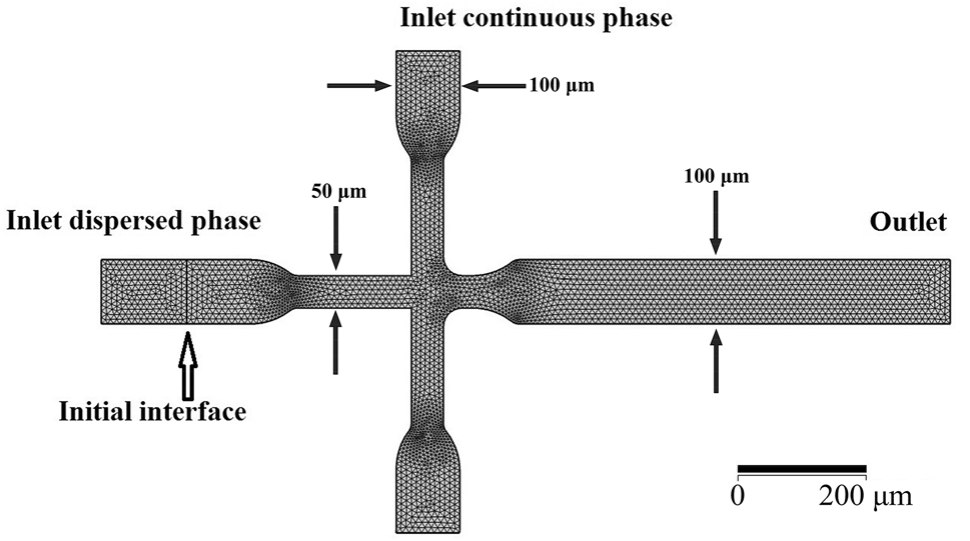
The pattern of the MFFD applied in simulations: meshes and boundaries situations specified for the microfluidic droplet generatrix in the two-dimensions model.

The strategy developed in this study is based on the evaluation of computer simulation outcomes. Now, we investigated the physical and chemical properties of CS (CS) and doxorubicin (DOX), effective in the size of droplets and creation rate, which contains: (1) CS + DOX mixture concentration, (2) CS + DOX mixture, and Vegetable Oil velocities flow ratio. The pattern of the Microfluidics Flow-focusing Device (MFFD) and the velocity of both phases determine the size and production rate of droplets 9,19. The MFFD configuration is demonstrated schematically in Fig. 1 and the comprehensive sizes are compiled in Table 1, including CS + DOX and vegetable oil inlets, chip outlet, and the orifice where droplets are formed. The simulations results are combined to provide a comprehensive assessment of the process of creating microfluidic droplets.

**Table 1:** Point by point sizes of MFFD.

	Input length (µm)	Output length (µm)	Input breadth (µm)	Output breadth (µm)	Angle (degree)
Dispersed phase	280	180	100	50	90
Continuous phase	100	180	100	50	
Orifice	50	80	50	100	40
Output	–	670	100	100	0

## Materials and methods

The following substances were used in this study: medium molecular weight CS [deacetylation degree 85%, 190–310 kDa], acetic acid glacial [C_2_H_4_O_2_, 100% (v/v)], vegetable oil [corn oil, pure], sorbitan monooleate [non-ionic surfactant, span^®^80], sodium tripolyphosphate [Na_5_P_3_O_10_, extra pure (TPP)], sodium sulfate [Na_2_SO_4_, extra pure ], aqueous tetramethylammonium hydroxide solution [(CH3)4N(OH), 25% (v/v)], and DOX [C_27_H_29_NO_11_, chemotherapy medication] from Sigma-Aldrich. MCF-7 breast cancer cell line was purchased from Pasteur Institute of Iran.

### Simulation

#### Numerical method

The preservationist Level-Set (LS) technique developed by Olsson and Kreiss^35^ has been validated for recreating the droplet formation process. The traditionalist LS technique describes the characteristic Mass-Conservation downside of the conventional LS technique. The equation of LS is communicated as:

1 $$\frac{\partial \phi }{{\partial t}} + \mathop u\limits^{ \to } .\nabla \phi = \gamma \nabla .\left[ {\varepsilon_{LS} \nabla \phi - \phi \left( {1 - \phi } \right)\frac{\nabla \phi }{{\left| {\nabla \phi } \right|}}} \right]$$

where $\varepsilon_{LS}$ is an issue with characterizing the breadth of the change layer, and it is to take place as a moiety-limited area of the regular mesh dimension in the region passed by the droplet. The parameter of *γ* determines the extent of the re-introduction of solidification. It should be rigorously considered for each specific downside. At each point, *γ* is excessively small, the width of the limit probably will not be steady, and variance in *ϕ* can be expected for numerical solution irregularities.

On the other hand, tremendous amounts of γ can result in an off-base interface. The parameter *ϕ* is within the restricted region of zero to one; whenever *ϕ* is less than 0.5 (*ϕ* < 0.5), it is relevant to phase one; nevertheless, when *ϕ* is larger than 0.5 (*ϕ* > 0.5), it is relevant to phase two, the component of *ϕ* and ε is the combination parameters: ε describe the extent of the crossing point while *ϕ* proceed placidly from 0 to 1, and it must contain the uniform scale as the computational meshing size of the components where juncture is scattered.

The two liquids are incompressible and laminar streams. Density and Dynamic viscosity are expected to be steady for CS + DOX and vegetable oil phases. The incompressible Navier–Stokes equation and the advection of the LS function ϕ are characterized by the governing equations for CS + DOX and vegetable oil stream. In contrast, the continuity equation explains its advection.

2 $$\nabla \cdot u = 0$$

3 $$\rho \left( {\frac{\partial u}{{\partial t}} + u\nabla \cdot u} \right) = \nabla \cdot \left[ { - P{\rm I} + \mu \left( {\nabla \cdot u + \left( {\nabla \cdot u} \right)^{T} } \right)} \right] + \rho g + F_{st}$$

*ρ, μ*, and *P* are the liquid's density, dynamic viscosity, and pressure. Henceforth, *u* is the velocity vector. Furthermore, *t* and *g* are time and gravitational acceleration. Finally, *Fst* is the surface tension force, and I is the identity matrix. The density *ρ* and dynamic viscosity *μ* of fluid in Eq. (3) are smoothed by *ϕ *across the jointing.

4 $$\rho = \rho_{1} + \left( {\rho_{2} - \rho_{1} } \right)\phi$$

5 $$\mu = \mu_{1} + \left( {\mu_{2} - \mu_{1} } \right)\phi$$

The parameters of *μ1, μ2,* and *ρ1, ρ2* are the dynamic viscosity and density of phase one and phase two, respectively. The quantity of *F*_*st*_ (surface tension force) is estimated as:

6 $$F_{st} = \nabla T = \nabla .\left[ {\left( {\sigma \left( {{\rm I} - nn^{T} } \right)} \right)\delta } \right]$$

where *δ* is the Dirac delta function which is non-zero at the fluid fringe, and the surface traction is σ. Besides, *n* and I are the interface normal and the identity matrix, respectively. The *n* is defined as:

7 $$n = \frac{\nabla \phi }{{\left| {\nabla \phi } \right|}}$$

Furthermore, the *δ* is advanced by a conventional equation measured as:

8 $$\delta = 6\left| {\phi \left( {1 - \phi } \right)} \right|\left| {\nabla \phi } \right|$$

#### Simulation model

The two immiscible fluids utilized the simulation model to describe the configuration of a 2D MFFD droplet generator. The MFFD regularly faces the laminar fluid behavior because of their diminished dimension to the micrometer; consequently, the Level Set and Laminar Two-phase Flow modules were decided to compute which simulation model. The mathematical simulation model using the incompressible fluid with the laminar flow regime, the two-phase flow, and the Level Set interface is implemented through Computational Fluid Dynamics Module in COMSOL Multiphysics^®^ 5.4 software.

Toward the computer simulation, the two-dimension pattern was utilized to illustrate the MFFD. The free triangular mesh was defined at a step of 0.5 µm. Finally, 1,783,832 domains and 8,978 boundary elements were performed for the pattern. The CS + DOX mixture and vegetable oil were chosen for the liquid characteristics from *COMSOL Multiphysics*^*®*^. The vegetable oil and CS + DOX solution were continuous and dispersed phases, respectively. The components were indicated as an incompressible Newtonian fluid. The conduit inside walls was determined as a wetted wall condition with a constant contact angle for all the cases. The CS + DOX and vegetable oil properties are summarized in Table 2. The MFFD schematic chart and the boundaries determined as inlets and outlets ducts for CS + DOX and vegetable oil phases are illustrated in Fig. 1.

**Table 2:** The components properties utilized in this simulation.

Property	Density (kg/m^3^)	Dynamics viscosity (mPa s)	Contact angle (rad)	Surface tension (mN/m)
Vegetable oil	925.5	39.21	**–**	**–**
**CS + DOX**				
0.2% (w/v) + 13.75 (µg/ml)	1153.25	2.153	0.39756	53.13
0.5% (w/v) + 13.75 (µg/ml)	1158.65	7.952	0.40997	53.15
1.0% (w/v) + 13.75 (µg/ml)	1167.25	18.145	0.45216	53.16

The laminar flow level-set interface includes a discretional multiphysics coupling boundary characteristic, wetted wall; a particular characteristic that overrides the wall characteristic in the Laminar or Turbulent flow interface. Moreover, the No Flow feature in the Level Set interface is accessible for a laminar and turbulent regime with wall functions or self-operating wall treatment^36^.

The border situation of the wetted wall is appropriate for surfaces in communication with the liquid–liquid border, as well as adds the following boundary force to enforce the contact angle. While this border situation is applied, the liquid–liquid border can move along the surface. Boundary walls dictate the non-penetration condition in the laminar regime and increase frictional constraints as follows:

9 $$\begin{array}{*{20}c} u & . & {n_{wall} = 0} \\ \end{array}$$

10 $$F_{\theta } = \sigma \delta \left[ {n_{wall} .n - \cos \left( {\theta_{w} } \right)} \right]n - \frac{\mu }{\beta }u$$

11 $$n_{wall} .\left( {\varepsilon_{LS} \nabla \phi - \phi \left( {1 - \phi } \right)\frac{\nabla \phi }{{\left| {\nabla \phi } \right|}}} \right) = 0$$

Here *n*_*wall*_ and *θ*_*w*_ are normal of the wall and contact angle, and *β* is the slip length. For mathematical computations, an appropriate decision is considering *β* equal to *h*, where the mesh element size is h. The border situation does not produce the tangential velocity element to zero. Nevertheless, the extrapolated tangential velocity component is 0 at a range of *β* outside the wall^36^.

The Parallel Direct Sparse Solver Interface (PARDISO) was applied to the phase initialization and time-related studies. When the continuous and dispersed phase goes through a tight way, the CS + DOX phase stretches. Eventually, droplets break up downstream of the duct. The simulation interval was between 0 and 300 ms, and the time step was established at 0.5 ms. The velocities flow rate of vegetable oil and CS + DOX phases was regulated with an orderly velocity. At the same time, the pressure at the end of the duct was specified as zero with non-viscous stress.

### Drug delivery and biological assays

In order to evaluate the encapsulation efficiency and release profile of the drug from microparticles, we need two standard diagrams with isopropanol and phosphate-buffered saline (PBS) solvents, respectively. To draw these graphs, we first dissolved a certain amount of DOX in the desired solvent, read its absorption wavelength at different drug concentrations using a spectrophotometer with three repetitions, and drew standard curves of the drug in the desired solvent. We investigated and reported the encapsulation rate and drug release from nanoparticles using the standard curves. To assess the release profile of the drug-loaded into the nanoparticles, we needed to provide physiological conditions. First, a buffer that can create conditions similar to blood pH should be selected. For this purpose, phosphate-buffered saline (PBS) was selected at pH 7.4 and pH 4.5. PBS buffer solution was used as the base solution to prepare different pH solutions. To adjust the pH equal to 4.5, an aqueous solution of 2% (v/v) acetic acid was used. The DOX release from CS formulation was evaluated using dialysis bags in phosphate buffer for 48 h at 37 and 42 °C, and pH 7.4 and 4.5. Initially, a specific volume of nanoparticles containing the drug was poured into a cellulose dialysis bag. Then the buffer around the dialysis bag was collected at different times, and the fresh phosphate buffer was replaced with the same volume. The samples were analyzed using ultraviolet spectrophotometry at the maximum wavelength of the drug. The release rate of the drug was measured at different times according to the standard curve of DOX in PBS.

We used an MTT assay to evaluate the cytotoxicity of DOX. This technique is based on converting tetrazolium to formazan in MTT dye by cells' mitochondrial enzymes. Formazan crystals in the presence of DMSO or isopropyl show a specific purple color that could be read at 570 and 630 nm by using a plate reader. First, we made specific drug concentrations and treated the cultured cells. For this purpose, the cells were counted after passage and proliferation and loaded with 10,000 cells in each well of a 96-well plate. After 24 h of cell incubation, cancer cells and healthy cells were treated with specific concentrations of DOX. After 48 h of cell incubation, the drug's toxicity on the cells was determined by MTT assay and plate reader. Finally, we calculated the survival rate at different concentrations and reported the IC50 of the drug on cancer cells.

## Result and discussion

### Microdroplet generation

The MFFDs are designed to have inlets and outlet conduits for dispersed and immiscible fluid streams colliding at an intersection with each other. Then, the CS + DOX and vegetable oil fluid are constrained to stream through a restricted hole at the intersection of both phase bays. As a steady phase, vegetable oil uses compression flow and tension force due to oil viscosity, forcing CS + DOX to flow through a tight aperture that breaks inside or downstream of the district. Gladwell et al. in 2012s, The pressing factor slope through the forming fluid string of CS + DOX and the drain at the junction subsequent disfigure the droplets in the entrance of downstream orientation to prevail the surface tension as a droplet is made up^37^.

The CS + DOX fluid plug streams downstream inside the main channel, while the apex of the stream of the isolated stage congregates to the conclusion of the input, and the process is rehashed. Consequently, the flow of the CS + DOX gets to be slim and breaks into beads. De Menech et al., in 2008s, classified the droplet formation into three multiple categories: (1) Squeezing, (2) Dripping, and (3) Jetting^36^. The Squeezing and Dripping process are generally accomplished in microchannel fluids, where the droplet production is impressed by the environmental restriction of the duct walls. In the Squeezing period, the upstream stress force applies the initial effect on the formation of droplets. On the contrary, the size of the capillary number has merely a minor efficacy that can be dismissed^38^.

The range of droplets in the Dripping category is influenced by the balance formed by tensions between interfacial and viscous forces. The side of a cross-connection can produce droplets, in which two conduits of the vegetable oil phase are situated at both sides of the channel of the CS + DOX phase and are perpendicular together. Jetting conditions in microchannel fluid are rarely employed. This process happens at too many fluid flows or little interfacial traction.

The perpendicular ducts transport the vegetable oil phase, and the direct duct is the inlet for the CS + DOX liquid. These immiscible liquids impact the side of the connection of the conduit entrance. In the first step of droplet generation, the flow of the CS + DOX interpenetrates into the hole, which is situated at the entry of the main channel, and the droplet commences to grow in dimension.

Subsequently, the droplet is steered downstream of the main duct by the pressure gradient and velocity flow. This development of droplet configuration could be separated into four levels: (1) Lag level, (2) Filling level, (3) Necking level, and finally (4) Detachment.

**Figure 2:**
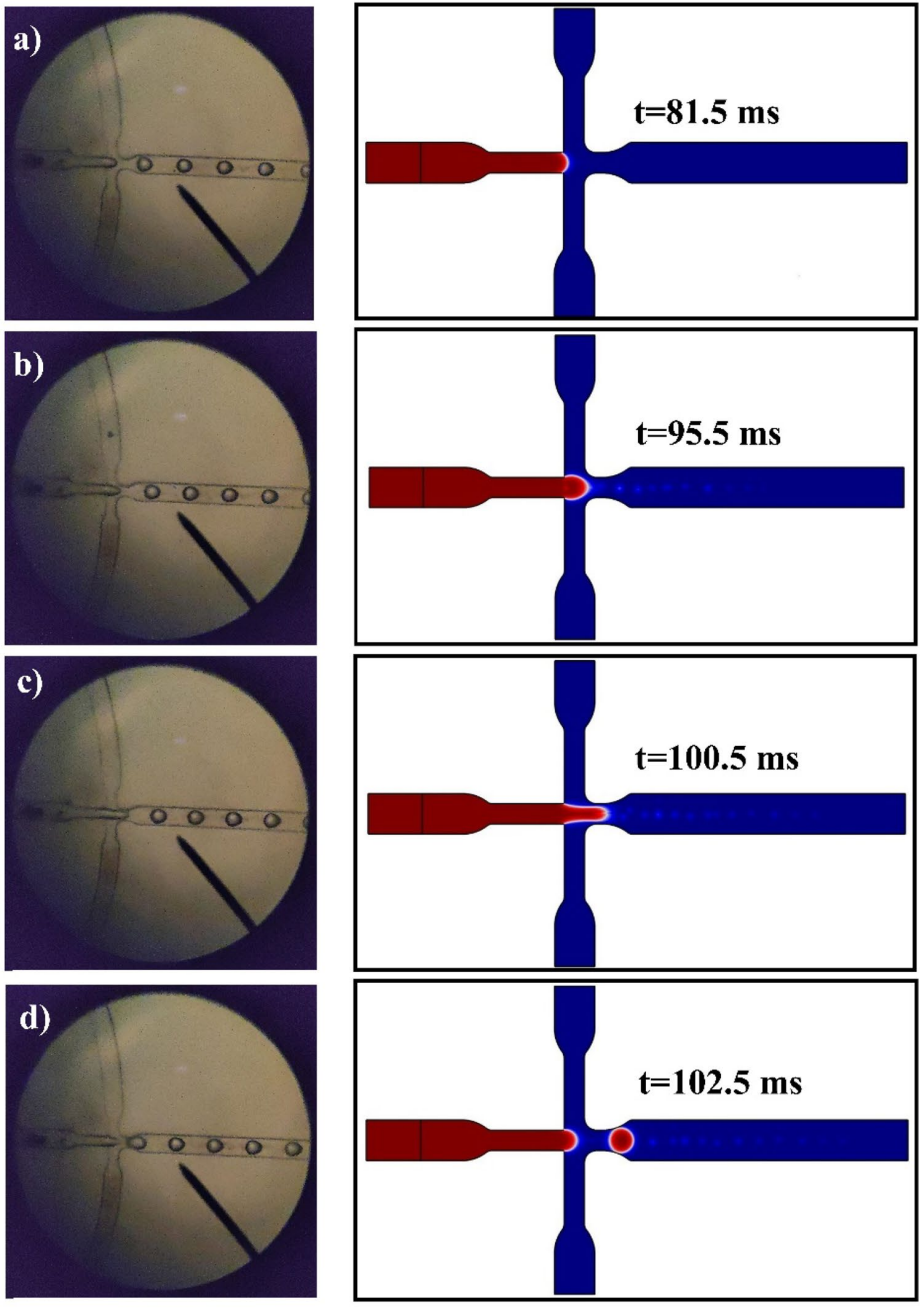
The microscopy and two-dimensional images of mechanism formation of CS + DOX microgel droplet injunction, (**a**) lag stage, (**b**) filling stage, (**c**) necking stage, (**d**) detachment. The CS + DOX and vegetable oil phase velocities were adjusted at 3.3 and 11.1 mm/s. The Concentration of CS and DOX are 0.2% and 13.75 (µg/ml), respectively.

Numerical simulation provides accessibility to all of the dynamic parameters of the pattern. Droplets configuration by detecting the pressure interdependence on time can be observed in the stable liquid straight away downstream of the center of the hole entrance. As shown in Fig. 2, The suite of snapshots demonstrates droplet configuration as a subordinate of time with concentrations of CS and DOX at 0.2% and 13.75 (µg/ml), respectively.

The experimental part of this study is as follows: First, design and fabricate the pattern of microfluidic flow-focusing upon silicon wafer by soft lithography techniques. Later, casting the mold of microfluidic flow-focusing with PDMS and bonding the PDMS mold of chip pattern upon slide glass by oxygen plasma. The CS + DOX and vegetable oil phases were injected into the chip by applying two dozing pumps.

The CS + DOX microgel droplet production mechanism with 0.2 wt % and 13.75 (µg/ml) of CS and DOC, respectively, is shown in Fig. 2.

**Figure 3:**
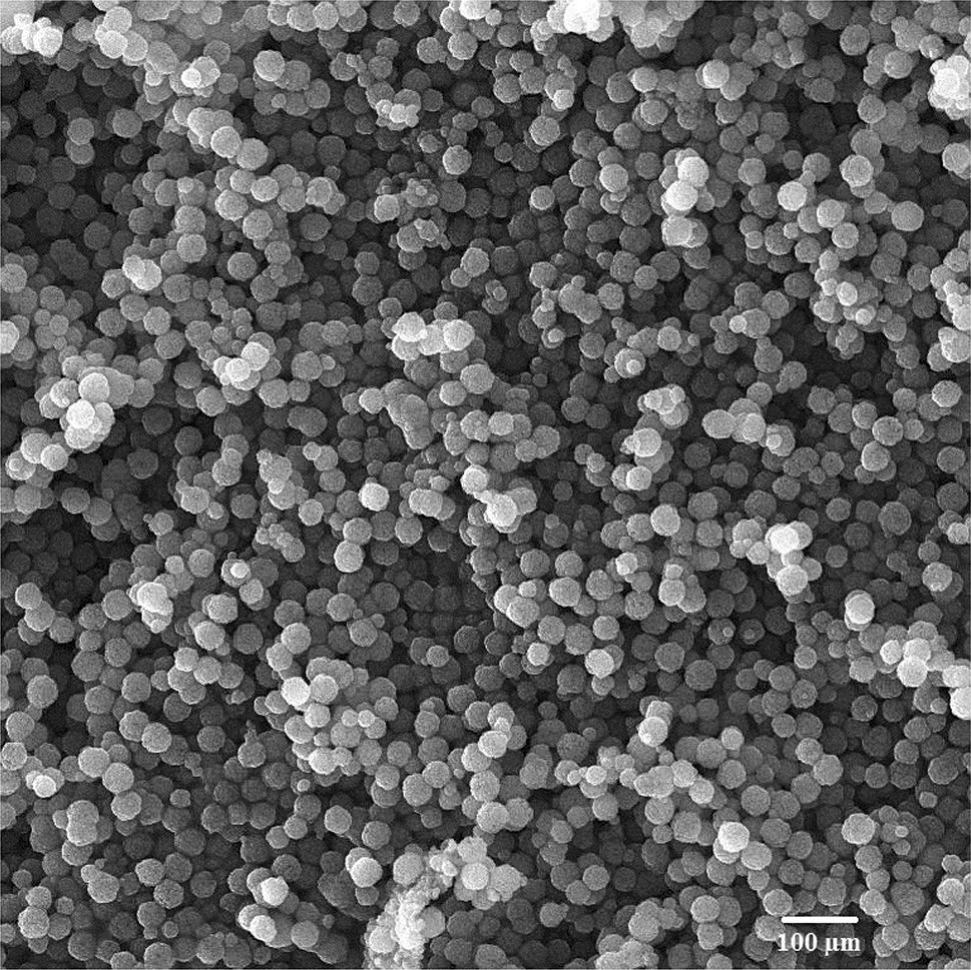
The CS with a concentration of 0.2% and 13.75 mg of DOX per ml of CS solution. The scanning electron microscope (SEM) of outcome CS + DOX microgel droplet of experimental result that the volumetric flow rate of CS + DOX phase was 3.3 mm/s and the volumetric flow rate of oil phase was 11.1 mm/s.

A 50–50 solution of TPP (7.5%wt) and Sodium sulfate (5.0% wt) is used as a crosslinker, poured into a microtube. The chip's output enters this solution, giving enough time to make cross-connections to the microgels. The aqueous and oil phases are separated. The oil phase is drained, and then the aqueous phase is washed with n-hexane and then with ethanol at 8000 rpm for 10 min and repeated three times. Figure 3 illustrates the SEM image of the CS + DOX microgel droplet.

**Figure 4:**
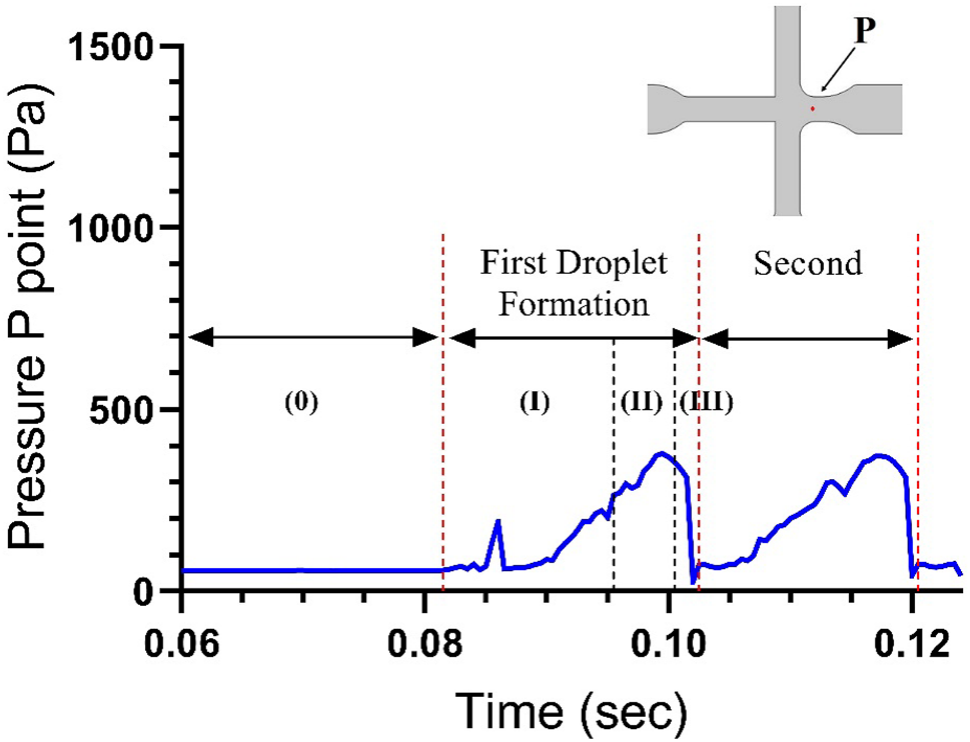
The procedure of the pressure slope at the P point for the duration of droplet configuration. The P point decides the point situated at the main duct entry. It reverberates the development of the droplet formation process. Three steps formation of droplet: (I) Lag, (II) Filling, and (III) Necking. The CS + DOX velocity is stable at 3.3 mm/s, and the velocity of vegetable oil is equal to 11.1 mm/s.

The P point shows pressure vacillations at the entry of the orifice hole as a function of the time for creating the CS + DOX liquid string is given in Fig. 4.

This diagram outlines that the pressure slope increases mildly up to the droplet's inception to yield beneath the pressure and shear force be formed and then diminution up to the dissociation. The division of the droplet is distinct by the sharp apex.

The pressure diminishes regularly during the second droplet formation time at the P point. Subsequently, the filament is still rising in dimension, and the P point pressure inception to soar. Commonly, in the formation period of a droplet, the pressure is more significant than before and after that. Additionally, the droplet's inner pressure is more than that of the stable phase in the circumfluent.

**Figure 5:**
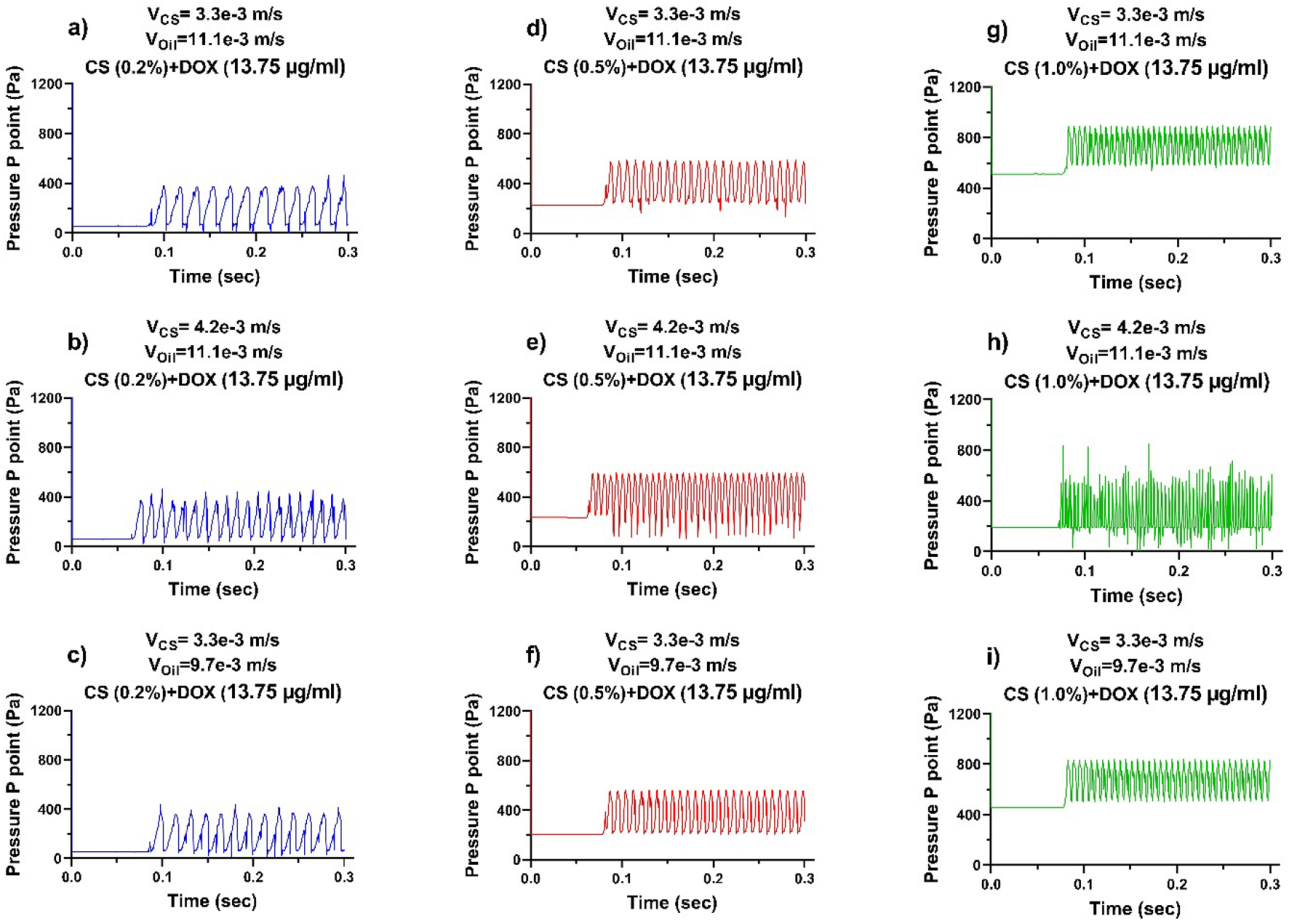
The computer simulation results in the pressure gradient at the P point. Impact of velocities and concentrations of the CS-DOX upon the droplet formation. The velocities of vegetable oil are changeable at 9.7 and 11.1 mm/s, and the CS-DOX velocities are regulated at 3.3 and 4.2 mm/s. The concentration of CS is (**a–c**) 0.2%, (**d–f**) 0.5%, **(g–i)** 1.0% (w/v), and also the concentration of DOX is 1.375% (w/v).

Similarly, the dynamic demeanour of the pressure slope at the spot of P point was appraised to three differences of the CS + DOX concentration (0.2%, 0.5%, 1.0% [w/v], and 13.75 [µg/ml]). The velocities range of CS + DOX was adjusted between 3.3 to 9.2 mm/sec. On the other hand, the velocity ratio of CS + DOX to vegetable oil was considered between 0.2 and 6, as shown in Fig. 5.

The impressive forces at the time of droplet formation, are pressure force, shear stress force and surface tensile force. The surface tensile force resists dispersed phase deformation, which is in contrast to the pressure and shear stress forces. When the amount of pressure force and shear stress is more than the surface tensile force, the droplet begins to thicken and thin. According to Table 2, increasing the concentration of chitosan affects the physical properties of the chitosan solution. These properties affect pressure forces, shear stresses and surface tension. As can be seen in Fig. 5, with an increasing in the concentration of chitosan at the same velocities of both phases, pressure curves become more fluctuate (from left to right).

On the other hand, according to Table 3 and Eqs. (2) and (3), by changing the ratio of disprtesed phase to continuous phase velocities (V_CS_/V_oil_), its effect is more on the pressure parameter. As a result, the force balance between pressure force and shear stress with surface tension, becomes more unstable and causes changes in the amplitude of oscillations. One droplet is produced from each oscillation of pressure, so the number of oscillations per unit of time and amplitude of the oscillation have a direct effect on the size and number of droplets produced as shown in Fig. 4.

**Table 3:** The amplitude of pressure fluctuations.

CS + DOX concentration		V_CS_/V_Oil_	Amplitude pressure (Pa)		
			0.2% (w/v) + 13.75 (µg/ml)	0.5% (w/v) + 13.75 (µg/ml)	1.0% (w/v) + 13.75 (µg/ml)
CS + DOX (mm/s)	3.3	0.297	305	330	310
Vegetable oil (mm/sec)	11.1				
CS + DOX (mm/s)	4.2	0.378	305	350	390
Vegetable oil (mm/s)	11.1				
CS + DOX (mm/s)	3.3	0.340	300	340	330
Vegetable oil (mm/s)	9.7				

Figure 5.a-i illustrates the pressure fluctuations as a function of the CS + DOX concentration. So by increasing the CS + DOX concentration, pressure fluctuations at the point P are also escalated, which sends back the effect of viscosity of the CS + DOX flow in the vegetable oil flow. Moreover, the amplitude of pressure fluctuations escalates from 320 to 380 Pa by increasing the CS + DOX concentration (see Fig. 5a,d,g). The amplitude of pressure fluctuations at the P point for each sub-figures of Fig. 5, is presented in Table 3.

On the other hand, the number of fluctuations in the unit of time is directly related to the ratios of CS + DOX and oil velocities, which increases with rising this ratio. The velocity ratio of CS + DOX to vegetable oil at concentrations of 0.2%, 0.5%, and 1.0% in Fig. 5a–c is 0.297, 0.378, and 0.340, respectively, which shows the dependence of pressure oscillations on velocity ratios. The pressure fluctuation escalated by increasing the velocity ratio and decreasing the velocity ratio. There is a remarkable pressure drop in the CS + DOX streamflow (94.6, 89.1, and 66.67%).

### Effect of fluid velocities ratio and CS + DOX concentration on diameter and creation rate of droplets

In the subsequent stage of the simulation study, the consequence of the velocities flow rate of both liquid and CS + DOX concentration was explored on the droplet diameter and creation rate.

In conformity with the previous research, the droplet size is indicated by an equilibrium between shearing force and interfacial tension^39^. The Capillary number (Ca), which is the ratio of the viscose force to the surface traction, has been applied to state this stress equilibrium^40^. The Capillary number C_a_ is calculated with:

12 $$C_{a} = \frac{{\mu_{C} u_{C} }}{\sigma } = \frac{{\mu_{C} F_{C} }}{s\sigma }$$

where *μ*_*C*_ and *u*_*C*_ are the dynamic viscosity and velocity of vegetable oil flow (Pa ·s and mm/s), and *F*_*C*_ is the volumetric flow rate of vegetable oil (µL/s). The surface area of the conduit is *s* (mm^2^). The interfacial traction is σ at the bursting moment (mN/m).

The less interfacial traction σ steers to the minuscule size of the droplet and the higher the formation periodicity.

Generally, the Reynolds number describes the liquid flow in a microfluidic chip. The Reynolds number is calculated by:

13 $${\text{Re}} = \frac{\rho ul}{\mu }$$

where *ρ*, *u,* and *μ* are fluid's density, velocity, and dynamics viscosity, respectively. *D* is the hydraulic diameter of the conduit. At Re <  < 1, the viscous force has the greatest effect on the behavior of the liquid. In conflict with this, the pressure inertial special efficacy profile is neglected; hence it could be identified from the direction of liquid particles precisely^21^.

This discovery has to steer to several requirements, including exposure to live cells to non-stop and gradual variations of bioactive molecules or temperature ratio, high-throughput broadcast, and lab-on-chip immunoassays^41^. Simulations were implemented at the various velocities of CS + DOX and vegetable oil and sundry CS + DOX concentrations; therefore, the droplets dimension size (d) was calculated (the droplets size was calculated by ImageJ software from the exported volume fraction graphical images of COMSOL simulation results). These variations were examined for the CS + DOX (*Q*_*cs*_) and vegetable oil (*Q*_*vo*_) phase velocity flow rate in the restricted area of 3.3–9.2 and 1.4–11.1 mm/s, respectively.

**Figure 6:**
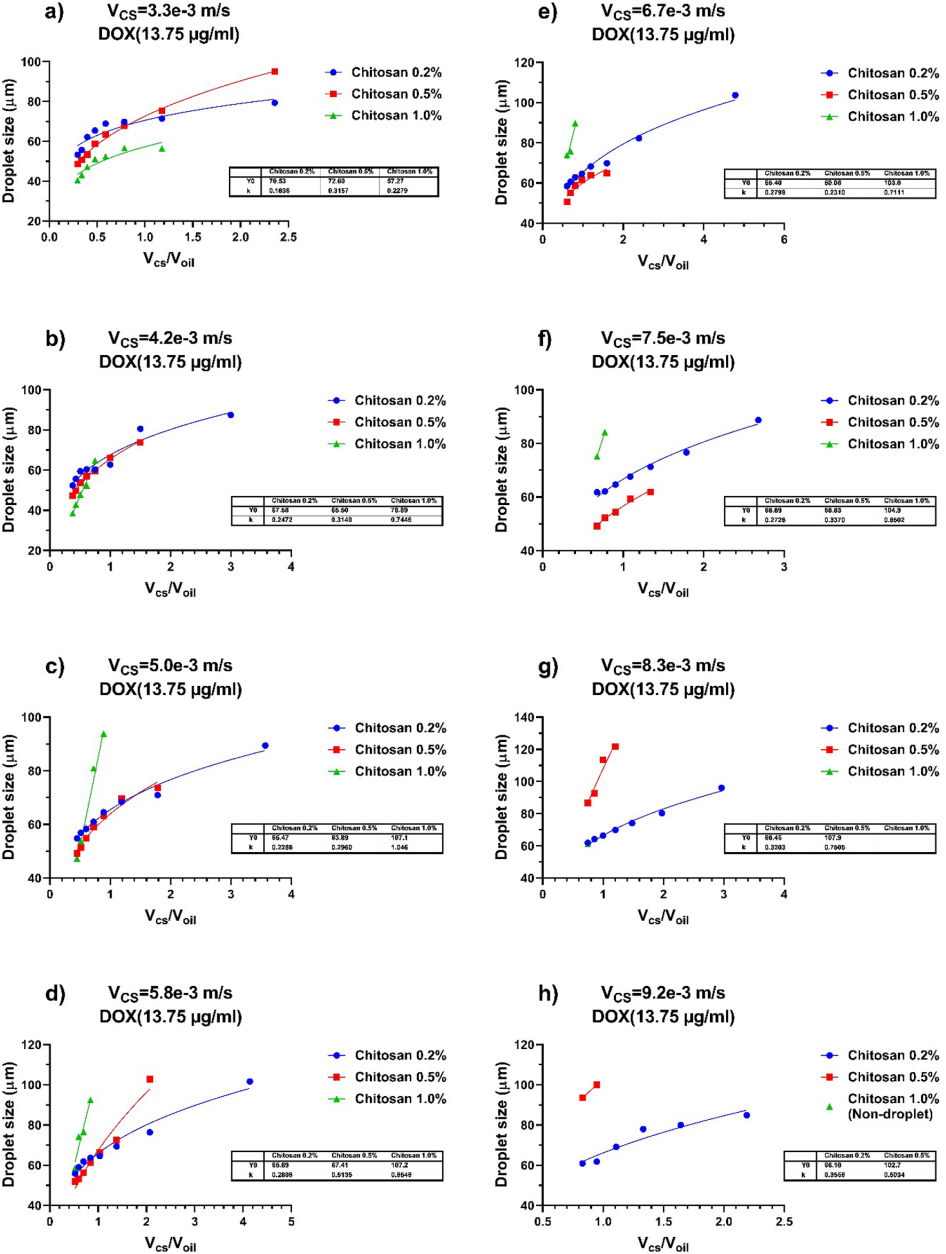
The dependence of the droplet size produced for each of the CS + DOX concentration to the variations of the velocity ratio of the dispersed phase to the continuous phase. The flow rate of vegetable oil and CS + DOX were selected at the limited area of 1.4 to 11.1 and 3.3 to 9.2 mm/s, respectively. The concentrations of CS are 0.2%, 0.5%, and 1.0% (w/v), and also the concentration of DOX is 1.375% (w/v). For each of subfigure, the CS + DOX velocity is fixed and the flow rate of the vegetable oli varies from 1.4 to 11.1 mm/s. The CS + DOX flow rates are (**a**) 3.3 mm/s, (**b**) 4.2 mm/s, (**c**) 5.0 mm/s, (**d**) 5.8 mm/s, (**e**) 6.7 mm/s, (**f**) 7.5 mm/s, (**g**) 8.3 mm/s and (**h**) 9.2 mm/s. As the CS + DOX flow rate increases, some points disappear for each of the three CS + DOX concentrations, indicating that no droplets are formed at this concentration and velocity ratio of the dispersed phase to the continuous phase. Continuous lines are curves plotted on experimental data using the power equation (Y = Y_0_ * X^k^). The values of Y_0_ and K of each of these curves are shown in the table in each subfigure.

A wide variety of droplet dimensions are apperceived that are interrelated to the CS + DOX and vegetable oil phase velocity flow rate and the concentration of CS + DOX (results shown in Fig. 6).

It is shown that the droplet diameter obtained for all concentrations of CS + DOX is in a similar inclination, and it is inclined to raise with the velocity of the vegetable oil flow rate declining. Figure 5 illustrates the droplets of CS + DOX with a different diameter to the velocity ratio flow rate of CS + DOX to vegetable oil.

It could be argued that the diameter of droplets decreases with rising the vegetable oil phase velocity (*Q*_*vo*_).

The variation in the extent of the consequential CS + DOX droplets size is evaluated as a parameter that depends on the CS + DOX concentration. As the concentration of CS + DOX increases, the amplitude of its changes also decreases. As well as the range of CS + DOX /vegetable oil velocities ratio in which droplets form is also reduced.

Variations in CS + DOX concentrations result in substantial droplet diameters, particularly at the lowest velocity of the vegetable oil flow. This could be described by the severity of significant turbulence in the attendance of junction, leading to rising the velocity of CS + DOX infusion into the vegetable oil flow and declining pressure drop.

Figure 6 shows the dependence of the droplet size produced based on the changes in CS + DOX concentration and the velocity ratio of the dispersed phase to the continuous phase. To show this dependence and the process of its changes, the line, the exponential and the power equations have been used to show this trend well. The results showed that the power equation has the best overlap and adaptation to the simulation data, and it has been used as a guideline for the eyes.

The surface tensile forces overcome the shear stress established by the fluid, so C_a_ values are less significant than 0.01. However, at amounts of C_a_ larger than 0.01 (C_a_ >  > 0.01), shear stresses perform a considerable duty^42^. If the velocity of CS + DOX fluid is significantly greater than the vegetable oil, the droplet was extended in the main channel and transformed into a filament shape. In the conflicting case, while the velocity ratios of both fluids have diminished, the CS + DOX droplet has not had sufficient time to stretch. Therefore, they developed and broke up without delay.

The velocity ratio of CS + DOX fluid, droplet size, and creation rates are employed as benchmarks to justify the acquiesce of the generated droplets.

The generation rate of droplets is well-thought-out, a semi-steady procedure where the input velocity ratio of both fluids is constant. Hence, the frequency of droplet generation is calculated by,

14 $$f_{droplet} = \frac{{Q_{d} }}{{D_{droplet} }}$$

Moreover, the number of droplets can be estimated from the following equation^43,44^:

15 $$No._{Droplet} = f_{droplet} \frac{{N_{frame} }}{F}$$

where *N*_*frame*_ is the total number of frames and *F* is the rate of each frame.

**Figure 7:**
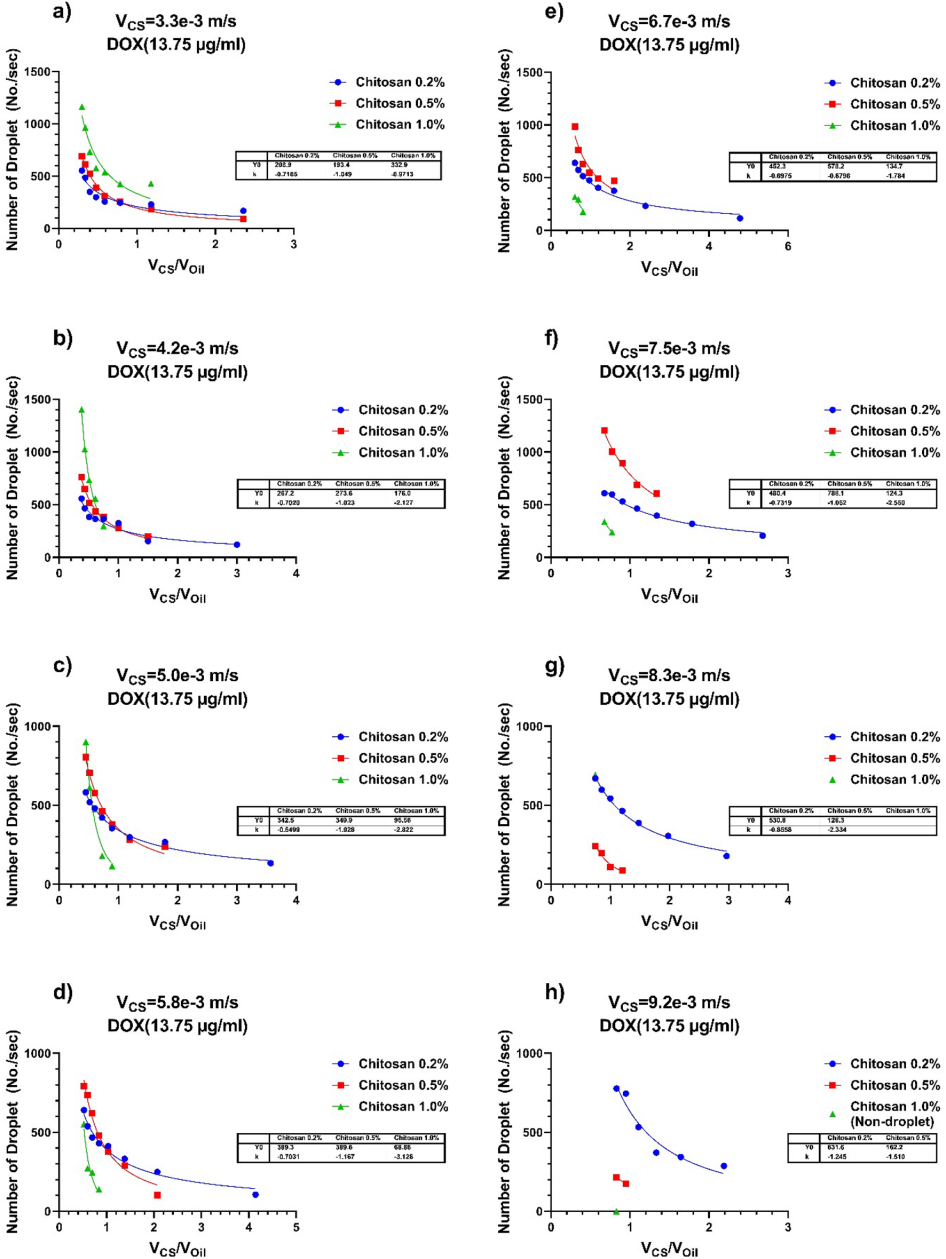
The consequence of velocity and concentration variations of CS-DOX and vegetable oil upon the number of droplet generation per unit time. The flow rate of vegetable oil and CS + DOX were selected at the limited area of 1.4 to 11.1 and 3.3 to 9.2 mm/s, respectively. The concentrations of CS are 0.2%, 0.5% and 1.0% (w/v), and also the concentration of DOX is 1.375% (w/v). For each subfigure, the CS + DOX velocity is fixed and the flow rate of the vegetable oli varies from 1.4 to 11.1 mm/s. The CS + DOX flow rate is (**a**) 3.3 mm/s, (**b**) 4.2 mm/s, (**c**) 5.0 mm/s, (**d**) 5.8 mm/s, (**e**) 6.7 mm/s, (**f**) 7.5 mm/s, (**g**) 8.3 mm/s and (**h**) 9.2 mm/s. Continuous lines are curves plotted on experimental data using the power equation (Y = Y_0_ * X^k^). The values of Y_0_ and K of each of these curves are shown in the table in each subfigure.

Figure 7 demonstrates the influence of vegetable oil's various velocity flow rates on the number of droplets formed at the adjusted CS + DOX velocity flow rate for 0.2%, 0.5%, and 1.0% concentrations of CS + DOX.

Figure 7a shows that the production rate of droplets for CS + DOX increases at a velocity rate of 3.3 mm/s as the concentration of CS + DOX increases. CS + DOX 1.0% produces a higher number of droplets than CS + DOX 0.2% and 0.5%. However, increasing the concentration leads to droplet production in a limited range of velocity ratios. With increasing CS + DOX velocity rate from 3.3 to 9.2, the droplet production range gradually decreases from 1%, 0.5%, and 0.2, respectively, until no more droplets are produced. It can be seen in Fig. 7a–h, CS + DOX with a concentration of 0.2%, had a different production process than other concentrations, so that had the highest droplet production rate with increasing dispersed phase velocity. However, for other concentrations, the droplet production rate decreased.

At a constant velocity of the CS + DOX fluid, the droplet size diminishes with increasing the velocity and flow rate of vegetable oil to achieve the range of droplet split-up at the junction. In addition, the droplet generation rate is also influenced by the flow rate of vegetable oil and the concentration of the dispersed phase. Equations (14) and (15) can be used to calculate the droplet diameter that correlates with its generation rate per unit of time.

Therefore, by increasing the dimension of the droplets in the streamflow of two immiscible fluids, the generation rate of the droplet diminishes, as shown in Fig. 7. Also, the diameter of droplets was reduced by increasing droplet production per unit time.

So, several factors may contribute to the droplet generation rate effectiveness, including turbulence strength enhancement, pressure drop near the junction, liquid flow obstruction, and increasing velocity of vegetable oil fluid.

Also, Fig. 7 shows the dependence of the number of droplets produced per unit of time based on the changes in CS + DOX concentration and the velocity ratio of the dispersed phase to the continuous phase. To show this dependence and the process of its changes, the line, the exponential and the power equations have been used to show this trend well. the results showed that the power equation has the best overlap and adaptation to the simulation data, and it has been used as a guideline for the eyes.

## Experimental results and discussion

**Figure 8:**
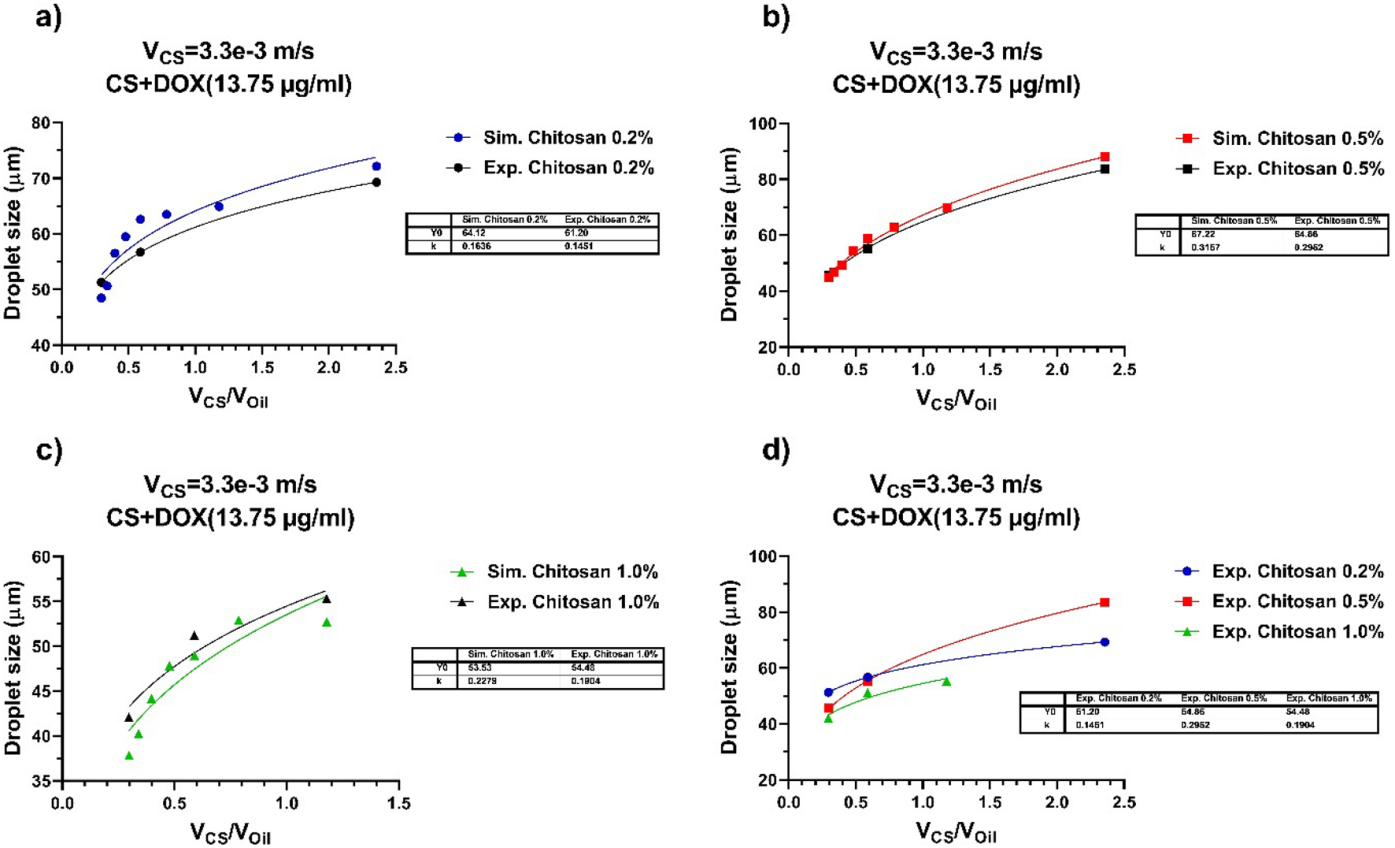
Comparisons the size of droplets generated using the computer simulator and experimental experiments. (**a**) CS 0.2%, (**b**) CS 0.5%, (**c**) CS 1.0%, (**d**) comparison of experimental data of droplet production for three concentrations of CS + DOX at the fixed velocity of the dispersed phase (3.3 mm/s), and velocity ratios of 2.36, 0.59 and 0.27, respectively. DOX concentration is 13.75 (µg/ml).

In the subsequent, three different concentrations of CS solution (0.2%, 0.5%, and 1.0% w/v) were prepared by supplementing the CS powder into 50 mL of aqueous acetic acid solvent (2%, v/v) in three glasses at room temperature and left nightly on a magnetic stirrer to get the pale-yellow homogeneous viscose CS solution. The residual CS powders, which had not dissolved, were removed using a syringe filter. A certain amount of the DOX drug per unit volume of CS solution (µg/ml) is combined with each of the CS solutions to finally develop a 1.375% (w/v) solution of the CS-DOX mixture. For this purpose, three glasses were mixed for 30 min at room temperature on a magnetic stirrer to reach a homogeneous mixture of CS and DOX. Vegetable oil and a mixture of CS + DOX with different concentrations were injected into the MFFD to generate droplets using two syringe pumps. The velocity rate of vegetable oil and CS + DOX mixture are selected based on software consequences. The vegetable oil velocities were adjusted at 11.1, 5.6, and 1.4 mm/s. Furthermore, the CS + DOX mixture velocity was set at 3.3 mm/s. As shown in Fig. 8, the experimental outcome of the CS-DOX mixture was compared with the computer simulation outcome, and acceptable compliance was apperceived between the simulation and experimental outcome. It can be noticed that in the 1.0% CS + DOX mixture, the size of the droplet was decreased compared to the other two concentrations. The dependence of droplet diameter on viscosity is shown in Fig. 8d.

According to Table 2, with increasing the concentration of chitosan, its dynamic viscosity also increases. At a constant velocity using Eq. (10), the Reynolds number decreases with increasing viscosity. The energy loss coefficient equation in laminar flow, is given below. By decreasing the Reynolds number, the amount of energy loss coefficient increases. The result is an increase in pressure drop. As a result, the effective forces in droplet production, are changed that cause the production of droplets with different diameters.

### Drug release and biological properties

**Figure 9:**
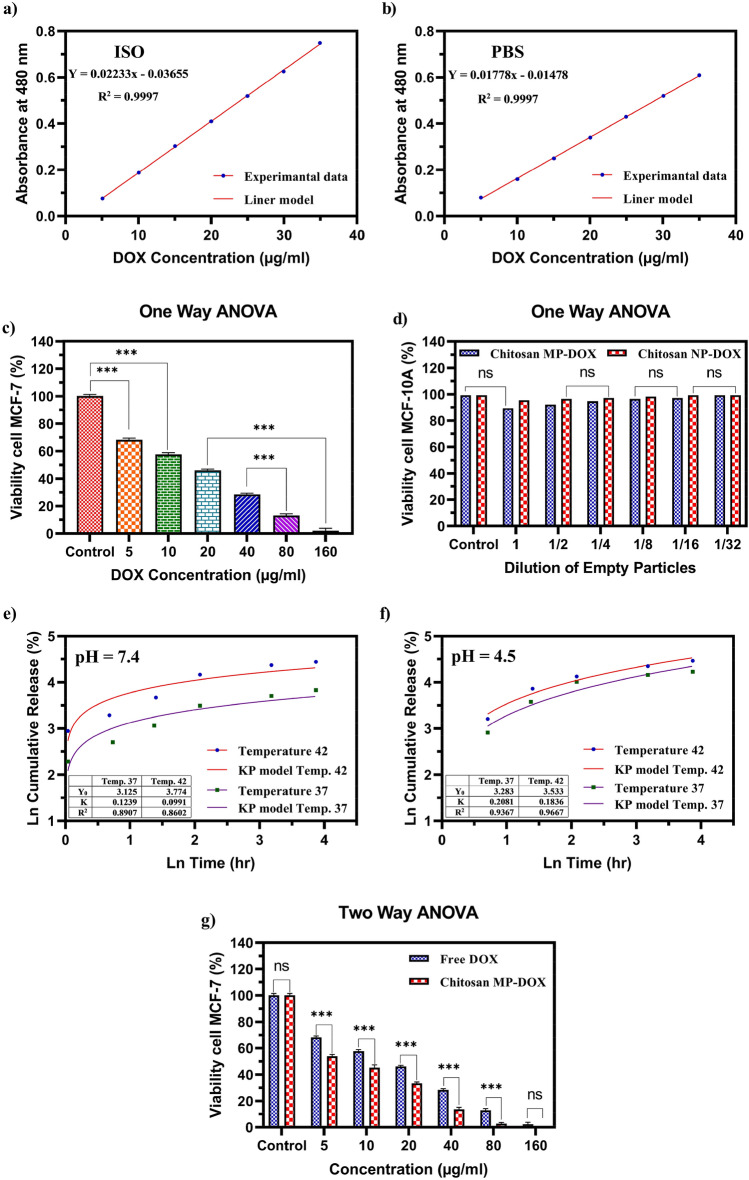
(**a**) Standard curve of DOX in ISO buffer at the wavelength of 480 nm. (**b**) Standard curve of DOX in PBS buffer at the wavelength of 480 nm. (**c**) Comparing the effect of the free form of DOX on MCF-7 breast cancer cell line in various concentrations. (**d**) The effect of blank chitosan particles at various diluents on HFF cells. e) Release profile kinetic of DOX from CS particles at various temperatures at pH  7.4. (**f**) Release kinetics of DOX from CS particles at various temperatures at pH  4.5. (**g**) Comparing the effect of the free form of DOX and CS-DOX on MCF-7 breast cancer cells. *ns* no significant difference. ***: P-value < 0.05.

The release of DOX from the modified formulation of CS was investigated in vitro. The results of the release pattern of DOX from the optimal formulation at pH 7.4 and 4.5 and temperatures of 37 and 42 °C for 48 h are shown in Fig. 9. The release profile of DOX followed a two-phase pattern. In the first phase, a rapid release has occurred and, over time, entered the second phase, which had a slow release. This indicates that the CS microcarriers are semi-targeted delivery systems. The drug's release rate at 42 °C and an acidic pH was significantly higher than the rate at 37 °C and a neutral pH, which was crucial in the controlled release of the drug into the cancer cells. According to the previous explanations, the release rate of DOX from CS microparticles was investigated at two different 37 °C and 42 °C temperatures. However, previous studies have shown that CS particles show a much better release rate at lower pH. In this study, we investigated the release pattern of DOX from CS microparticles at pH 4.5 and 37 and 42 °C.

The Korsmeyer-Peppas model is used to study drug release from polymer composites. The equation of which is as follows:

16 $$F = \frac{{M_{t} }}{{M_{\infty } }} = K_{KP} t^{n}$$

where *F* is fraction of drug released at time t, *M*_*t*_ and *M*_*∞*_ are amount of drug released at time t and total amount of drug in dosage form, respectively. *K*_*KP*_ and t are kinetic constant and time respectively. n is diffiusion or release exponent which characterize the mechanism of release. So that if n = 1, the release is Zero-order, if n = 0.5, the release is best described by Fickian diffiusion and if 0.5 < n < 1, the release is through anomalous diffiusion.

As can be seen in Fig. 9 e–f, the drug is better released in a buffer solution of pH 4.5. Also, there is no significant difference between the temperature of 42 and 37 degrees in this pH. But this difference in buffer solution with pH 7.4, is very significant for two temperature conditions. On the other hand, experimental data for buffer solution with pH 4.5, are more compatible with the Korsmeyer-Peppas model.

The results showed that besides following a specific two-phase pattern, the drug release pattern had a relatively higher release rate at 37 °C and 42 °C, as shown in Fig. 9e–f.

**Figure 10:**
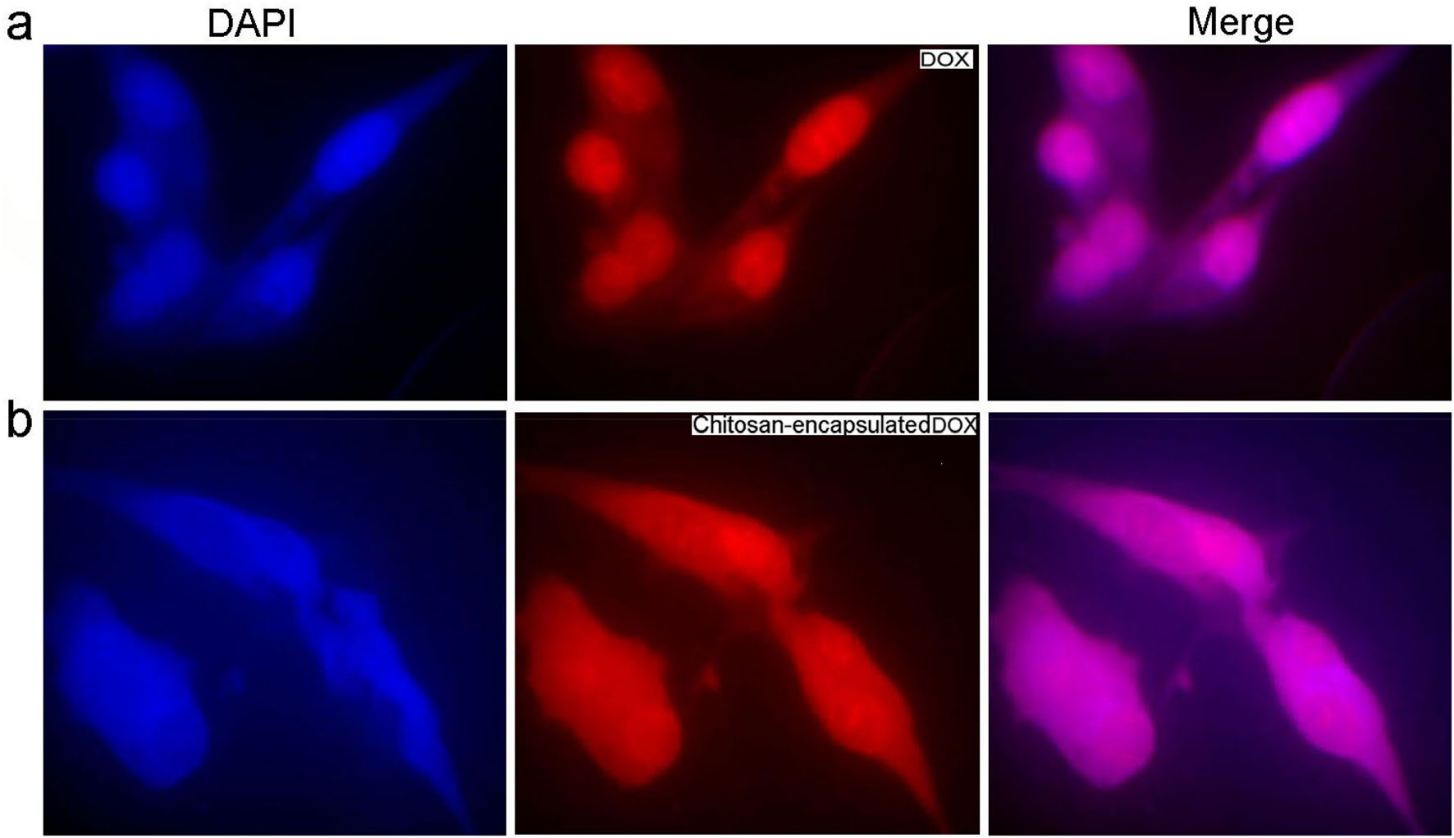
(**a**) Cellular uptake behavior of DOX by MCF-7 breast cancer cell line. (**b**) Cellular uptake behavior of CS-DOX by MCF-7 breast cancer cell line. DAPI is used to stain nuclei of cells. Due to its fluorescent nature, DOX causes the cell cytoplasm to turn red on the fluorescent imaging. The images confirm the uptake of the drug by the cells.

Results of the MTT assay showed that the toxicity of the encapsulated DOX had a greater effect on cancer cells than the free drug (Figs. 9, 10). Normal and cancerous cells were treated with specific concentrations of the drug (5, 10, 20, 40, 80, 160 µg/ml) for 48 h and the results showed a significant relationship (P < 0.05) between the toxicity of free drug (IC50 = 13.75 g/ml) and the encapsulated drug (IC50 = 7.143 g/ml). The survival rate of mcf-7 cells exposed to free DOX, and microparticles containing DOX for 48 h, was evaluated by assessing the significance of cytotoxicity at different drug concentrations based on the ANOVA test and reported according to P statistical criteria (Fig. 9). Also, the cytotoxicity of CS particles in the form of nanoparticles and microparticles on normal human breast cell line (MCF-10A) was evaluated and compared, which showed that the CS particles in both nano- and micro-states had the lowest cytotoxicity on the normal cells.

A cellular uptake study was performed to compare and investigate free and encapsulated DOX uptake in 3 h on an MCF-7 cell line. In this technique, DAPI was used to stain nuclei of cells by sticking to the rich A-T region of DNA. Thus, the nucleus of cells in the images obtained by fluorescent microscopy is blue. On the other hand, because of its fluorescent nature, DOX is red in fluorescent imaging. Figure 10 indicates the uptake of CS particles containing DOX by the cells. The red color illustrates the DOX uptake by cells. The blue color also indicates the nucleolus of cancerous cells. The mixture of these colors expresses the behavior uptake of the particles by cells. According to the standard curves of DOX in isopropanol buffer, the loading rate of DOX in CS particles was investigated. The loading rate of the drug in nanoparticles was 93%. Also, the maximum amount of drug released from the particles at various temperatures and pH conditions in 48 h was reported.

It is clear that DOX alone is more toxic than CS/DOX carrier, because CS/DOX carrier releases the drug slowly (controlled release). Therefore, the carrier is more biocompatible for healthy cells than free DOX. However, in some drug delivery systems, the inhibitory effects of the drugs-loaded nanocarriers are more those that of free drugs.

## Conclusion

A computational fluid dynamic model was exploited for deep understanding of the CS droplet size and formation in a FF microchannel. The simulation results represented an alternate approach to reach visions into the complicated process. The role of channel geometries, channel aspect ratio and flow rate ratio on the CS droplet charactristics, were fully described. The experimental research demonstrated the promising potential of the CS microparticles for biomedical applications especially in drug delivery. Considering the CS characteristics such as excellent bioactivity, biodegradability, and biocompatibility, regulating and qualifying the CS droplets in a microchip is very important for different purposes, like biosensing, cell/tissue engineering, drug delivery, drug screening lab-on-a-chip, organ-on-a-chip, 3D cultures, and bioimaging. Concisely, a comprehensive simulation study was carried out to exhibit the best protocol, platform, and design to reach desired amount/size/morphology of CS droplets for many applications in microfluidic-based chips/devices, as well as to investigate the mechanism of droplet formation and generation with tuneable shape/size, addressing several challenges biomedical applications of monodispersed CS microparticles.

## Data Availability

The datasets used and/or analyzed during the current study are available from the corresponding author on reasonable request.
